# The Mediating Role of Lean Soft Tissue in the Relationship between Somatic Maturation and Bone Density in Adolescent Practitioners and Non-Practitioners of Sports

**DOI:** 10.3390/ijerph18063008

**Published:** 2021-03-15

**Authors:** Ricardo R. Agostinete, André O. Werneck, Santiago Maillane-Vanegas, Luis Gracia-Marco, Esther Ubago-Guisado, Annie M. Constable, Romulo A. Fernandes, Dimitris Vlachopoulos

**Affiliations:** 1 Laboratory of InVestigation in Exercise (LIVE), Department of Physical Education, Sao Paulo State University (UNESP), Presidente Prudente 19060-900, Brazil; santiagovanegas16@gmail.com (S.M.-V.); romulo.a.fernandes@unesp.br (R.A.F.); 2 Center of Epidemiological Research in Nutrition and Health, Department of Nutrition, School of Public Health, University of São Paulo, São Paulo 01246-904, Brazil; andreowerneck@gmail.com; 3 PROFITH “PROmoting FITness and Health through Physical Activity”, Research Group, Sport and Health University Research Institute (iMUDS), Departament of Physical Education and Sport, Faculty of Sport Sciences, University of Granada, 18071 Granada, Spain; lgracia@ugr.es (L.G.-M.); esther.ubago@gmail.com (E.U.-G.); 4 Instituto de Investigación Biosanitaria ibs GRANADA, 18012 Granada, Spain; 5 Escuela Andaluza de Salud Pública (EASP), 18011 Granada, Spain; 6 Children’s Health and Exercise Research Centre, Sport and Health Sciences, University of Exeter, Exeter EX1 1TX, UK; ac857@exeter.ac.uk (A.M.C.); D.Vlachopoulos@exeter.ac.uk (D.V.); 7 Institute of Biomedicine, Kuopio Campus, University of Eastern Finland, FI-70211 Kuopio, Finland

**Keywords:** bone health, youth, team sport, exercise

## Abstract

This study aimed to identify the mediating effect of lean soft tissue (LST) in the association between somatic maturation and areal bone mineral density (aBMD) in adolescents by sex and sport participation. The sample included 558 adolescents (401 males, mean age of 14.0 years) that were practitioners of sports (11 sport modalities, *n* = 402) and a non-sport group (*n* = 157). Somatic maturation was assessed by using a validated peak height velocity prediction equation. Dual-energy X-ray absorptiometry (DXA) was used to assess aBMD (upper and lower limbs, spine and total body less head—TBLH) and LST. For both sexes, LST mediated the association between somatic maturation and aBMD at all skeletal sites (mediation percentage ranging from 36.3% to 75.4%). For sport and non-sport groups, the LST also mediated the association between somatic maturation and aBMD at all skeletal sites (mediation percentage ranging from 51.6% to 85.6%). The direct effect was observed in all groups, except for lower limbs and TBLH in the non-sport group. The association between somatic maturation and aBMD was mediated by LST in adolescents of both sexes and regardless of involvement in organized sports. Our findings highlighted the role of improving LST to mitigate the association of somatic maturation with aBMD.

## 1. Introduction

During human growth, biological maturation affects all tissues of the human body, including lean soft tissue (LST) (muscle tissue) and adipose tissue [1]. Biological maturation also affects the bone tissue through bone mass accrual [1] and bone fracture incidence [2,3]. LST has an important metabolic, locomotive and physiological role with areal bone mineral density (aBMD), mainly due to mechanotransduction forming a “bone–muscle unit” [4]. Bone tissue has important functions of support, movement, protection, blood cell production and homeostatic regulation of minerals [5], while LST is also responsible for locomotion, stability and is the major repository of protein [6]. These variables are in effect especially during adolescence, when several morphological and functional changes occur due to maturation events [1], including peak height velocity (PHV). PHV is a common landmark of somatic maturation that is considered as the period of maximum growth in stature. The PHV is especially important for bone tissue considering that during the circumpubertal years (−2 to +2 years from PHV), around 33–46% of the total bone mass observed in adulthood is acquired [7]. Moreover, bone mineral content (BMC) and areal bone mineral density (aBMD) are positively related to somatic maturation in both cross-sectional [8] and longitudinal [9] studies.

Among the determinants of bone outcomes in adolescents, the literature has shown that LST is the strongest predictor of aBMD [10] due to the osteogenic effect of muscle contractions on bone [11], causing strains and mechanical loading of muscle on bones during body movements. Moreover, LST is closely related to the biological maturation process. It is known that the peak accrual of lean mass follows the PHV and precedes the peak accrual of bone mass [12]. Thus, LST is an important determinant of aBMD. In this sense, LST could be considered a potential mechanism underlying the association between biological maturation and aBMD [12]. 

Different studies assessing the association between biological maturation and bone aspects are limited in that only the direct association between timing and status of maturation on bone variables was assessed [13,14,15]. However, the potential mediating role of LST in the association between somatic maturation, an indicator of biological maturation, and aBMD has not been investigated in adolescents who practice sports. Moreover, there is a lack of evidence on which covariates are more appropriate in bone studies in pediatric populations; somatic maturation, LST or both. 

The association among somatic maturation, LST and aBMD is particularly important for adolescent practitioners of sports because LST plays an important, mediating role in the association between physical fitness and bone outcomes [16]. Besides that, sports participation involves a great number of muscle contractions, which generate forces and mechanical loads responsible for the bone adaptations [17]. Thus, the aim of this study was to examine the mediating role of LST on the relationship between somatic maturation and aBMD in adolescents considering the sex and sport participation. 

## 2. Materials and Methods 

### 2.1. Sample

This cross-sectional study is part of a large study (Analysis of Behaviors of Children During Growth–ABCD Growth Study) from the Laboratory of InVestigation in Exercise (LIVE) that was conducted in the city of Presidente Prudente (Brazil) from October 2013 to May 2017. 

At the beginning of the study, a cooperation agreement was established between the LIVE and local authorities. After explaining the proposal to private/public schools and sports institutions, the researchers were given formal authorization to start data collection and adolescents were regularly registered and invited to be part of the research. To participate in the study, adolescents were required to provide a written consent form signed by a parent or guardian. The Ethical Board of the São Paulo State University (UNESP) approved the investigation (Process numbers 1.677.938/2016 and 02891112.6.0000.5402).

The sample was composed of 558 adolescents (401 males) with a mean age of 14.1 years (±2.0). The non-sport group included 156 adolescents (109 males) while the sport group included 402 adolescents, divided as follows: basketball (*n* = 53 males), soccer (*n* = 101 males), swimming (*n* = 58 (males *n* = 40)), volleyball (*n* = 33 (males *n* = 2)), karate (*n* = 36 (males *n* = 19)), judo (*n* = 48 (males *n* = 30)), kung fu (*n* = 30 (males *n* = 21)), baseball (*n* = 12 males), gymnastics (*n* = 10 females), tennis (*n* = 6 males) and track and field (*n* = 15 (males *n* = 9)). 

The inclusion criteria to be eligible were (1) chronological age between 10 and 18 years; (2) a minimum of 6 months of participation in the specific sport (sport group); (3) a minimum engagement of three or more hours per week (sport group); (4) non-involvement in other organized sports (sport groups); (5) the absence of engagement in organized sport (presence of coach, training routine and competitions (non-sport group)); (6) the consent forms were signed by parents and guardians of the participants. 

### 2.2. Body Composition Measures

Dual-energy X-ray absorptiometry (DXA) (Lunar DPX-NT; General Electric Healthcare, Little Chalfont, Buckinghamshire, UK) with GE Medical System Lunar software (version 4.7) was used to obtain areal bone mineral density (aBMD, g/cm^2^), lean soft tissue (LST, kg) (sum of all soft tissue, bone-free and fat-free) and fat mass (kg), measured at the university laboratory in a temperature-controlled room. A trained researcher performed all scans and tested the scanner quality before the first exam of each day. The scans were performed using a standardized protocol with the participants remaining in the supine position and wearing only light clothing, without shoes. Regional analysis for aBMD of upper limbs, lower limbs, spine and total body less head (TBLH) occurred off-line after the scans took place [18]. The coefficient of variation for this device was 0.66% (in total-body aBMD analysis, *n* = 30 participants not involved in this study), setting the lines (region of interest-roi) in the segments (upper limbs, lower limbs and spine) as requested for the General Electric Healthcare company and stated in previous studies [19]. 

### 2.3. Anthropometry

A stadiometer (Sanny, model American Medical of the Brazil Ltd., Brazil; accurate to 0.1 cm) was used to measure height and sitting height to later allow an estimate of leg length and subsequent maturity offset. An electronic scale (Filizzola PL 150, model Filizzola Ltd., Brazil; with a precision of 0.1 kg) was used to obtain body mass. The measures were assessed using standardized techniques described in the literature [20] and by a trained researcher. 

### 2.4. Somatic Maturation

Anthropometric measurements (body mass and stature) were used to estimate years from the peak height velocity (PHV) through mathematical formulas predicted by Moore et al. [21]. This measure denotes the distance (in years) from the PHV, a landmark of somatic maturation that is considered the period of maximum growth in stature. Negative values denote that the individual has not yet passed the PHV, and positive values denote that the individual has already passed the PHV. The coefficient of determination has been reported in the literature (r^2^ = 0.898, Standard error of the estimate-SEE = 0.5 in females and r^2^ = 0.896, SEE = 0.5 in males) [21]. 

### 2.5. Statistical Analysis 

**Figure 1:**
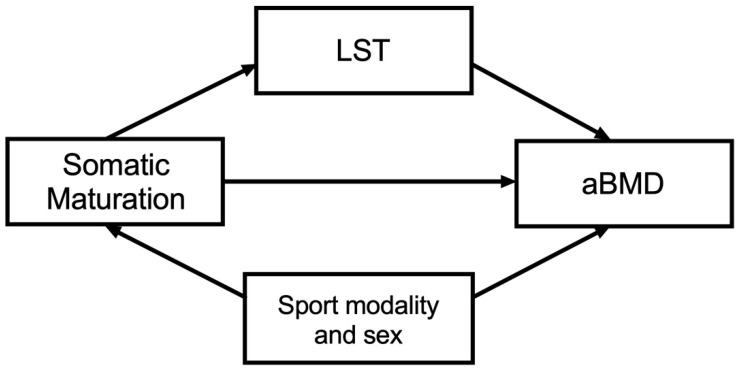
Theoretical model. Independent variable: somatic maturation; dependent variable: areal bone mineral density (aBMD); mediator variable: lean soft tissue (LST). Confounders: sex and sport modality.

Characteristics of the sample were expressed as mean values, standard deviation (SD) and 95% confidence intervals (95% CI). Sex and sport participation differences in descriptive variables were tested using the Mann–Whitney test. Pearson correlation coefficients were used to find associations between the independent, mediator and dependent variables. For mediation models, we used the model proposed by Valeri and Vanderweele [22]. The theoretical model is presented in Figure 1. In this method, the total effect (i.e., the effect of somatic maturation on aBMD) is decomposed into controlled direct effects (i.e., the direct effect of somatic maturation on aBMD through pathways not related to LST), reference interaction (i.e., the effect of somatic maturation on aBMD due to the interaction with LST), mediated interaction (i.e., the effect of somatic maturation on aBMD due to both mediation by and interaction with LST) and pure indirect effects (i.e., the effect of somatic maturation on aBMD mediated by LST). All the correlation and mediation models were adjusted for the same variables, aiming to reduce the potential role of confounders. Considering that there is variability in aBMD, to control for the engagement in different types of sports [23], all the analyses were adjusted by sport modality, aiming to control for variations due to sport. We acknowledge that this will not physiologically remove the effect of different sports, which we could not control, but it removes the statistical differences in aBMD to allow us to perform the specific analyses. Thus, the analysis by sex was adjusted by sport modality, while analysis by sport participation was adjusted by sex and sport modality. The command “med4way” on Stata 15.1 was used [24] and the level of significance was set at *p* < 0.05.

## 3. Results

Table 1 presents the characteristics of the sample. In the crude analysis by sex, females presented an advanced somatic maturation (1.76 ± 1.67 years in females vs. 0.69 ± 1.71 years in males) as well as lower LST (33.7 ± 5.4 kg in females vs. 45.5 ± 11.3 kg in males) and aBMD (g/cm^2^) at all body sites (upper limbs: 0.766 ± 0.091 in females vs. 0.813 ± 0.140 in males; lower limbs: 1.166 ± 0.118 in females vs. 1.286 ± 0.205 in males and TBLH: 0.955 ± 0.100 in females vs. 1.061 ± 0.155 in males), except in the spine (1.078 ± 0.151 g/cm^2^ in females vs. 1.025 ± 0.173 in males). In the analysis by sport participation, the groups presented the same somatic maturation stage (*p* = 0.555). The sport group had a higher LST (43.7 ± 11.6 kg in sport group vs. 38.3 ± 9.7 kg in non-sport group) and aBMD at all body sites (upper limbs: 0.813 ± 0.134 in sport group vs. 0.764 ± 0.112 in non-sport group; lower limbs: 1.279 ± 0.196 in sport group vs. 1.182 ± 1.164 in non-sport group; spine: 1.056 ± 0.162 in sport group vs. 0.997 ± 0.177 in non-sport group and TBLH: 1.063 ± 0.146 in sport group vs. 0.989 ± 0.127 in non-sport group.) 

Table 2 presents the partial correlations between exposure, mediator and outcomes by sex (adjusted by sport modality) and sport participation (adjusted by sex and sport modality). In all analyses by sex and sport participation, somatic maturation presented a strong positive correlation with LST (*r* = 0.593 to 0.920) as well as aBMD at all skeletal sites (*r* = 0.554 to 0.820). Moreover, LST presented a strong positive correlation with aBMD at all skeletal sites (*r* = 0.609 to 0.863) in males, females, the sport group and the non-sport group.

**Figure 2:**
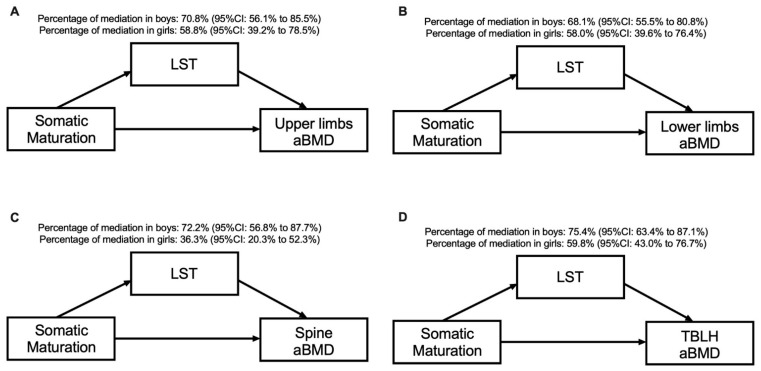
Mediation models of LST in the relationship between somatic maturation and aBMD sites according to sex and after adjusting for sport modality.

Table 3 and Figure 2 present the sex-specific mediation effects of LST in the association between somatic maturation and aBMD at different skeletal sites. The significant variables in the correlation analysis were inserted in the mediation models. In all models, for both sexes, LST mediated the association between somatic maturation and aBMD in the upper limbs (70.8% for boys and 58.8% for girls), lower limbs (68.1% for boys and 58.0% for girls), spine (72.2% for boys and 36.3% for girls) and TBLH (75.4% for boys and 59.8% for girls). The direct effect remained significant for all aBMD variables and in both sexes (*p* > 0.05). 

**Figure 3:**
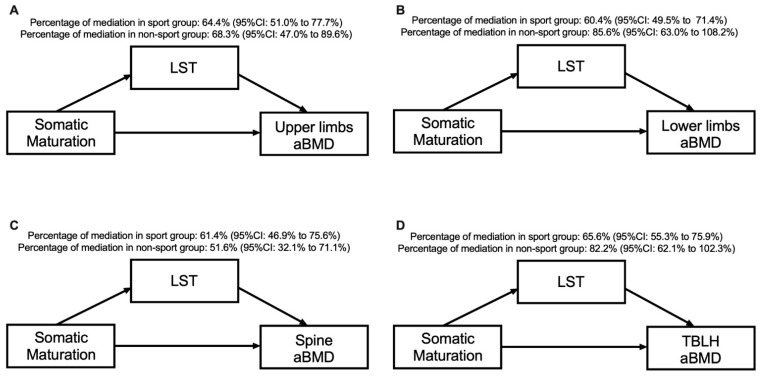
Mediation models of LST in the relationship between somatic maturation and aBMD sites according to sport participation status and after adjusting for sport modality and sex.

Table 4 and Figure 3 present the mediation models by sport participation. Our results show that for both the sport and non-sport groups, LST mediated the association between somatic maturation and aBMD in the upper limbs (64.4% for sport group and 68.3% for non-sport group), lower limbs (60.4% for sport group and 85.6% for non-sport group), spine (61.4% for sport group and 51.6% for non-sport group) and TBLH (65.6% for sport group and 82.2% for non-sport group). There was a direct effect of somatic maturation on all aBMD variables in the sport and non-sport groups, except for the lower limbs (*p* = 0.239) and TBLH (*p* = 0.106) in the non-sport group. 

## 4. Discussion

This study examined the mediating role of LST in the relationship between somatic maturation and aBMD in adolescents, stratified by sex and by sport participation. The main findings were that LST mediated the association between somatic maturation and aBMD at all skeletal sites (upper limbs, lower limbs, spine and TBLH) in male and female adolescents regardless of sport participation. Furthermore, the direct association between somatic maturation and aBMD remained significant after accounting for LST as a mediating variable, except for lower limbs and TBLH in the non-sport group. 

This study created a theoretical model assessing the mediating role of LST on the association between somatic maturation and aBMD. In the analysis by sex, our results showed that the LST mediated the association between somatic maturation and aBMD at all skeletal sites in both sexes. Therefore, somatic maturation influences aBMD by influencing LST, which then influences aBMD through the mechanical load applied in the bone matrix, consequently increasing the recruitment of osteogenic cells [25]. This is apparent in males and females, which corroborates a previous theory suggesting that bone adapts according to the necessity of adaptation caused by external factors in males and females, including greater LST [26]. 

Our findings support those from the study by Jackowski et al. [12] and by Vlachopoulos et al. [10] which found that muscle variables were a significant predictor of bone outcomes, including aBMD. In the study by Jackowski et al., the authors also found, in the Saskatchewan Pediatric Bone Mineral Accrual Study, that the mechanical loading of muscle contractions causes bone adaptations and that the peak of lean tissue precedes the peak of bone mineral content accrual, following the maturation sequence to attain PHV and peak tissue (peak height velocity, peak lean mass, peak weight velocity and peak fat mass/peak of bone mass, simultaneously, in that order) [27,28], indicating a possible causality of this association. Therefore, biological maturation is associated with gains in LST, which, theoretically, precede and are partially responsible for the gains in bone mineral density in males and females [12]. 

Boys showed a higher percentage of mediation by LST at all skeletal sites (68.1% to 75.4% in boys vs. 36.3% to 59.8% in girls). In fact, it is well documented that males have higher LST than females mainly during adolescence and maintained in adulthood [29], which would justify this percentage, as well as higher values of aBMD in males observed in the literature. Furthermore, in females, body fat is more important in the prediction of aBMD than in males, mainly in the spine [30,31] (skeletal site with lower percentage of mediation by LST in the study in females), partially due to the synthesis of estrogen in the adipose tissue, which promotes cortical and trabecular bone accrual [32].

The mediating effect of LST in the association between somatic maturation and aBMD at all skeletal sites was also observed in both the sport and non-sport groups, reinforcing the positive effect of LST on aBMD explained above. For this reason, LST is usually used as a covariate in studies focusing on bone, growth and sport participation [33]. However, there was no direct effect on the lower limbs and total body in the non-sport group. Despite it being established in the literature that adolescents who practice sports have greater muscle recruitment and more weight-bearing activities, which enhance aBMD gain [23,34,35,36,37,38,39], it is possible that sports participation can modify the association between somatic maturation and aBMD by other mechanisms beyond LST, such as hormonal [40], while non-sport practitioners’ aBMD at these skeletal sites may depend on LST to a greater extent compared to sport practitioners.

Another possible explanation refers to the fact that the magnitude of the total, direct and indirect effects of somatic maturation with aBMD is lower in the non-sport group compared to the sport group, which could explain the higher percentage of mediation by LST in this group. This indicates a possible greater effect of sport participation in the association between somatic maturation and aBMD, both directly and indirectly through LST. However, more studies, particularly longitudinal, are needed to verify this mediation effect. 

This study has practical implications such as showing the importance of applying strategies for the development of LST (muscle tissue) in order to enhance the gains of aBMD derived from the maturation process. Besides that, taking into account that biological maturation is considered an important confounder in growth studies of bone health among sports sciences [27], these findings reinforce the importance for studies involving aBMD variables in adolescents to consider both somatic maturation and LST as important predictors and confounding variables. 

Limitations of this study include that it did not adjust the analyses by habitual physical activity and nutritional status, which are potential confounders [10,41]. In addition, our sample included adolescents between 10 and 18 years of age, and the indicator of somatic maturation might have a bias of estimation in samples with a large age range (outside the optimal band of estimation) [42]. In addition, despite the adjustment by sport modality, we acknowledge that sports affect aBMD differently, and for this reason, studies analyzing each sport modality separately should be encouraged. Lastly, similar analyses involving adolescents from different countries investigating sex differences should be encouraged due to the differences in the growth and development of children and adolescents in developed and developing countries [43]. In contrast, the strengths of the study include that it included a large sample of more than 600 adolescents with indicators of objectively measured aBMD and LST and reliable assessment of somatic maturation.

## 5. Conclusions

In summary, the present study shows that LST mediates the relationship between somatic maturation and aBMD of different skeletal sites in male and female adolescents, regardless of involvement in organized sports. These findings suggest that the potential mediating effect of LST in the association between somatic maturation and aBMD is similar in males and females; however, the mediation magnitude seems to be affected by sport participation in the lower limbs and TBLH. Finally, our findings highlight the role of improving LST to mitigate the association of somatic maturation with aBMD and future studies should be encouraged in order to understand other potential mediators of this association. 

## Figures and Tables

**Table 1:** Characteristics of the sample according to sex and sport participation (*n* = 558).

Total (*n* = 559)	Males (*n* = 401) Mean ± SD	Females (*n* = 157) Mean ± SD	*p*
Chronological age, years	14.2 ± 2.0	14.0 ± 2.0	0.336
Somatic maturation *, years	0.69 ± 1.71	1.76 ± 1.67	<0.001
Age at PHV, years	13.5 ± 0.6	12.3 ± 0.6	<0.001
Body mass, kg	60.1 ± 15.3	53.1 ± 12.1	<0.001
Stature, cm	168.4 ± 12.4	159.5 ± 8.9	<0.001
LST, kg	45.5 ± 11.3	33.7 ± 5.4	<0.001
Body fat, kg	10.8 ± 8.4	16.3 ± 8.7	<0.001
Upper limbs aBMD, g/cm^2^	0.813 ± 0.140	0.766 ± 0.091	0.003
Lower limbs aBMD, g/cm^2^	1.286 ± 0.205	1.166 ± 0.118	<0.001
Spine aBMD, g/cm^2^	1.025 ± 0.173	1.078 ± 0.151	<0.001
TBLH aBMD, g/cm^2^	1.061 ± 0.155	0.995 ± 0.100	<0.001
	Sport Group (*n* = 402)	Non-sport Group (*n* = 156)	
Chronological age, years	14.0 ± 1.9	14.4 ± 2.4	0.088
Somatic maturation *, years	0.97 ± 1.68	1.06 ± 1.97	0.555
Age at PHV, years	13.1 ± 0.8	13.3 ± 0.9	<0.001
Body mass, kg	59.4 ± 14.8	54.8 ± 14.2	0.001
Stature, cm	166.9 ± 12.3	163.4 ± 11.8	0.004
LST, kg	43.7 ± 11.6	38.3 ± 9.7	<0.001
Body fat, kg	12.1 ± 8.9	12.9 ± 8.8	0.381
Upper limbs aBMD, g/cm^2^	0.813 ± 0.134	0.764 ± 0.112	<0.001
Lower limbs aBMD, g/cm^2^	1.279 ± 0.196	1.182 ± 0.164	<0.001
Spine aBMD, g/cm^2^	1.056 ± 0.162	0.997 ± 0.177	<0.001
TBLH aBMD, g/cm^2^	1.063 ± 0.146	0.989 ± 0.127	<0.001

PHV, peak of height velocity; aBMD, areal bone mineral density; LST, lean soft tissue. *, years from PHV; TBLH, total body less head.

**Table 2:** Partial correlations of independent, mediator and dependent variables according to sex and sport participation.

	LST	Upper Limbs aBMD	Lower Limbs aBMD	Spine aBMD	TBLH aBMD
	*r*/*p*-Value	*r*/*p*-Value	*r*/*p*-Value	*r*/*p*-Value	*r*/*p*-Value
**Somatic Maturation**					
Males	0.898/<0.001	0.731/<0.001	0.818/<0.001	0.744/<0.001	0.820/0.001
Females	0.593/<0.001	0.554/<0.001	0.576/<0.001	0.619/<0.001	0.599/<0.001
Sport group	0.920/<0.001	0.690/<0.001	0.792/<0.001	0.680/<0.001	0.781/<0.001
Non-sport group	0.813/<0.001	0.648/0.001	0.647/<0.001	0.719/<0.001	0.665/<0.001
**LST**					
Males	--	0.770/<0.001	0.840/<0.001	0.771/<0.001	0.863/<0.001
Females	--	0.683/<0.001	0.704/<0.001	0.609/<0.001	0.744/<0.001
Sport group	--	0.681/<0.001	0.757/<0.001	0.647/<0.001	0.761/<0.001
Non-Sport group	--	0.700/<0.001	0.735/<0.001	0.701/<0.001	0.750/<0.001

Analysis by sex adjusted by sport modality. Analysis by sport participation adjusted by sex and sport modality. aBMD, areal bone mineral density; PHV, peak height velocity; LST, lean soft tissue; TBLH, total body less head.

**Table 3:** Mediation models of the association between somatic maturation and aBMD by LST according to sex.

	Total Effect	Controlled Direct Effect	Reference Interaction	Mediated Interaction	Pure Indirect Effect
**aBMD Males**					
Upper limbs	**0.00602 (0.00548 to 0.00657)**	**0.00176 (0.00081 to 0.00271)**	−0.00005 (−0.00011 to 0.00053)	**0.00005 (0.00003 to 0.00007)**	**0.00427 (0.00343 to 0.00510)**
Lower limbs	**0.00892 (0.00821 to 0.00963)**	**0.00284 (0.00163 to 0.00406)**	−0.00002 (−0.00006 to 0.00002)	0.00002 (−0.00001 to 0.00005)	**0.00608 (0.00500 to 0.00716)**
Spine	**0.00720 (0.00653 to 0.00788)**	**0.00200 (0.00081 to 0.00319)**	−0.00001 (−0.00004 to 0.00002)	0.00001 (−0.00002 to 0.00004)	**0.00520 (0.00416 to 0.00625)**
TBLH	**0.00690 (0.00637 to 0.00743)**	**0.00170 (0.00084 to 0.00256)**	−0.00002 (−0.00006 to 0.00083)	0.00002 (0.000002 to 0.00005)	**0.00520 (0.00442 to 0.00598)**
**aBMD Females**					
Upper limbs	**0.00304 (0.00233 to 0.00376)**	**0.00125 (0.00049 to 0.00201)**	0.00001 (−0.00003 to 0.00004)	−0.00001 (−0.00003 to 0.00001)	**0.00179 (0.00120 to 0.00238)**
Lower limbs	**0.00409 (0.00317 to 0.00501)**	**0.00172 (0.00076 to 0.00267)**	0.00001 (−0.00003 to 0.00005)	−0.00001 (−0.00003 to 0.00001)	**0.00237 (0.00162 to 0.00313)**
Spine	**0.00558 (0.00447 to 0.00670)**	**0.00356 (0.00227 to 0.00484)**	0.00003 (−0.00007 to 0.00013)	−0.00003 (−0.00006 to 0.00001)	**0.00203 (0.00115 to 0.00290)**
TBLH	**0.00361 (0.00285 to 0.00436)**	**0.00145 (0.00069 to 0.00220)**	0.00001 (−0.00003 to 0.00005)	−0.00001 (−0.00003 to 0.00001)	**0.00216 (0.00152 to 0.00279)**

Analysis performed by sex and adjusted for sport modality. Significant values are in bold and the effect of somatic maturation on aBMD mediated by LST is presented in “Pure Indirect Effect”.

**Table 4:** Mediation models of the association between somatic maturation and aBMD by LST according to sports participation status.

	Total Effect	Controlled Direct Effect	Reference Interaction	Mediated Interaction	Pure Indirect Effect
**aBMD**					
**Sport group**					
Upper limbs	**0.00592 (0.00536 to 0.00648)**	**0.00211 (0.00123 to 0.00299)**	−0.00005 (−0.00010 to 0.00033)	**0.00005 (0.00002 to 0.00007)**	**0.00381 (0.00306 to 0.00456)**
Lower limbs	**0.00885 (0.00814 to 0.00957)**	**0.00350 (0.00241 to 0.00459)**	−0.00003 (−0.00007 to 0.00001)	0.00003 (−0.000003 to 0.00006)	**0.00535 (0.00441 to 0.00630)**
Spine	**0.00702 (0.00631 to 0.00773)**	**0.00271 (0.00156 to 0.00285)**	−0.000004 (−0.00004 to 0.00003)	0.000004 (−0.00005 to 0.00003)	**0.00431 (0.00335 to 0.00527)**
TBLH	**0.00688 (0.00634 to 0.00741)**	**0.00237 (0.00159 to 0.00315)**	−0.00002 (−0.00005 to 0.00001)	**0.00002 (0.000001 to 0.00004)**	**0.00451 (0.00381 to 0.00520)**
**Non-sport group**					
Upper limbs	**0.00041 (0.00034 to 0.00048)**	**0.00013 (0.00003 to 0.00023)**	−0.0000001 (−0.000002 to 0.000002)	0.0000001 (−0.0000002 to 0.0000003)	**0.00028 (0.00020 to 0.00036)**
Lower limbs	**0.00056 (0.00046 to 0.00066)**	0.00008 (−0.00005 to 0.00021)	0.0000002 (−0.000005 to 0.000006)	−0.0000002 (−0.000001 to 0.0000001)	**0.00048 (0.00036 to 0.00060)**
Spine	**0.00064 (0.00054 to 0.00074)**	**0.00031 (0.00016 to 0.00046)**	0.0000002 (−0.000006 to 0.000006)	0.0000002 (−0.0000006 to 0.0000001)	**0.00033 (0.00021 to 0.00045)**
TBLH	**0.00046 (0.00038 to 0.00054)**	0.00008 (−0.00002 to 0.00018)	0.0000001 (−0.000003 to 0.000003)	−0.0000001 (−0.0000004 to 0.0000001)	**0.00038 (0.00029 to 0.00047)**

Analysis performed by sport participation and adjusted for sex and sport modality. Significant values are in bold and the effect of somatic maturation on aBMD mediated by LST is presented in “Pure Indirect Effect”.

## Data Availability

Due to data privacy, the data presented in this study are available upon request from the corresponding author and Principal Investigator of the ABCD Growth study.
